# Is It Safe to Initiate/Optimize the Medication of HFrEF Patients During Hospitalization for Acute Decompensation?

**DOI:** 10.3390/jcm14082664

**Published:** 2025-04-13

**Authors:** Ruxandra Maria Christodorescu, Daniel Miron Brie, Alina Diduța Brie, Cristian Mornoș, Simona Ruxandra Drăgan, Constantin Tudor Luca, Dan Dărăbanțiu, Alexandru Tîrziu

**Affiliations:** 1 Department of Internal Medicine, “Victor Babes” University of Medicine and Pharmacy, Eftimie Murgu Square, No. 2, 300041 Timisoara, Romania; christodorescu.ruxandra@umft.ro; 2 Research Center of the Institute of Cardiovascular Diseases, Gheorghe Adam St., No. 13A, 300310 Timisoara, Romania; mornos.cristian@umft.ro (C.M.); simona.dragan@umft.ro (S.R.D.); constantin.luca@umft.ro (C.T.L.); 3 Cardiovascular Disease Institute Timisoara, Gheorghe Adam St., No. 13A, 300310 Timisoara, Romania; alexandru.tirziu@umft.ro; 4 Department of Cell and Molecular Biology, “Victor Babes” University of Medicine and Pharmacy, Tudor Vladimirescu Street, No. 14, 300174 Timisoara, Romania; alina.brie@umft.ro; 5 ANAPATMOL Research Center, “Victor Babes” University of Medicine and Pharmacy, Tudor Vladimirescu Street, No. 14, 300174 Timisoara, Romania; 6 Department of Cardiology, “Victor Babes” University of Medicine and Pharmacy, Eftimie Murgu Square, No. 2, 300041 Timisoara, Romania; 7 Arad County Emergency Clinical Hospital, Andrényi Károly Street, No. 2–4, 310037 Arad, Romania; 8 Department of Functional Sciences, “Victor Babes” University of Medicine and Pharmacy, Tudor Vladimirescu Street, No. 14, 300174 Timisoara, Romania

**Keywords:** heart failure with reduced ejection fraction (HFrEF), decompensated heart failure, four pillars of therapy, guideline-directed medical therapy (GDMT), acute heart failure hospitalization, natriuretic peptides, volume overload, diuretics, hypotension, hyperkalemia

## Abstract

**Background:** Current guidelines emphasize the importance of initiating or optimizing the four pillars of heart failure with reduced ejection fraction (HFrEF) therapy—beta-blockers (BB), mineralocorticoid receptor antagonists (MRA), angiotensin receptor–neprilysin inhibitors (ARNI), and sodium–glucose cotransporter-2 inhibitors (SGLT2i)—during hospitalization for acute decompensation. This study compares clinical characteristics and outcomes in HFrEF patients hospitalized for decompensated heart failure based on whether they were newly initiated on or were already receiving at least one of these four pillars. **Methods:** This prospective observational study included 203 HFrEF patients hospitalized for acute decompensation. Patients were divided into two groups: Group A (*n* = 126), not receiving any of the four pillars prior to admission, and Group B (*n* = 77), receiving at least one. Clinical and biological parameters were evaluated during hospitalization, with outcomes including changes in weight, blood pressure, heart rate, renal function (serum creatinine), electrolyte levels (sodium, potassium), and 30-day mortality. Statistical analyses included the non-parametric Mann–Whitney test and Chi-squared test. **Results:** Baseline characteristics (age, gender, LVEF, NT-proBNP) were similar between the two groups. No significant difference was observed in 30-day mortality (Group A: 7.14%, Group B: 5.55%, *p* = 0.74). Both groups experienced significant improvements in systolic and diastolic blood pressure and heart rate during hospitalization (*p* < 0.05). While serum creatinine levels remained stable in both groups, creatinine dynamics (Δcreatinine) were significantly different (*p* = 0.02), with Group B exhibiting a higher increase. The improvement in ejection fraction was more pronounced in Group A (*p* = 0.057) compared to Group B. Both groups demonstrated significant improvements in NYHA functional class (*p* < 0.001). In Group B, the use of MRAs and SGLT2 inhibitors significantly increased during hospitalization (*p* = 0.01 and *p* < 0.001, respectively). **Conclusions:** The initiation or optimization of the four pillars of HFrEF therapy during hospitalization for acute decompensation is feasible and well-tolerated. Early intervention leads to improvements in clinical parameters and functional status, supporting guideline recommendations for in-hospital initiation or optimization of HFrEF therapy. Special consideration should be given to renal function when optimizing therapy.

## 1. Introduction

In 2021, for the first time, heart failure (HF) societies worldwide proposed the universal definition of HF as a clinical syndrome characterized by signs and symptoms suggestive of pulmonary and/or systemic congestion (dyspnea, pitting edema, fatigue during decreasing levels of physical activity) caused by structural and/or functional cardiac abnormalities associated with elevated natriuretic peptide levels. From the same year, the classification of heart failure describes four types of HF based on the left ventricular ejection fraction (LVEF): (1) heart failure with preserved ejection fraction (HFpEF, LVEF > 50%); (2) heart failure with mildly reduced ejection fraction (HFmrEF, LVEF = 41–50%); (3) heart failure with reduced ejection fraction (HFrEF, LVEF < 40%); and (4) heart failure with improved ejection fraction (HFimpEF, symptomatic HF with a baseline LVEF ≤ 40%, a ≥10 percentage increase from baseline LVEF, and a second measurement of LVEF > 40%) [1].

According to recent heart failure guidelines (both European and American), the recommended treatment of heart failure with reduced ejection fraction (HFrEF) should be based on the four pillars: beta-blockers (BB), mineralocorticoid receptor antagonists (MRA), angiotensin receptor blockers–neprilysin inhibitors (ARNI) and sodium–glucose cotransporter type 2 inhibitors SGLT2i [2,3]. Diuretic treatment is indicated when there are signs of pulmonary or systemic congestion, but it does not reduce long-term mortality [4,5,6]. Therefore, initiating or optimizing therapeutic regimens to contain the four pillars (beta blockers, MRAs, SGLT2 inhibitors, and ARNI/ACE inhibitors or ARBs) as early as possible, preferably during hospitalization, is desired [7]. Optimizing treatment after an acute episode of decompensated HF was well tolerated and accepted by the patients, reduced symptoms, and improved the quality of life, as shown in a recent study [8]. In our study, we aimed to compare the clinical characteristics and outcomes of patients with decompensated heart failure who required hospital admission and did not have the four pillars in their treatment regimen compared to patients who had at least one of the four pillars treatment initiated. HF treatment was initiated or optimized during hospitalization after acute HF decompensation, and clinical and biological parameters were evaluated.

## 2. Methods

### 2.1. Study Design and Participants

This study was a prospective observational cohort analysis conducted in a county hospital to evaluate the clinical characteristics and outcomes of patients with heart failure with reduced ejection fraction (HFrEF) upon hospital admission. A total of 203 patients diagnosed with HFrEF were included in the study. Participants were stratified into two groups based on their medication regimens before admission: Group A included 126 patients with decompensated heart failure who were not receiving any of the four pillars of heart failure treatment before admission (angiotensin-converting enzyme inhibitors, angiotensin-receptor blockers, angiotensin-receptor/neprilysin inhibitors (ACEi/ARBs/ARNI), beta-blockers (BB), mineralocorticoid receptor antagonists (MRA), and sodium–glucose cotransporter-2 inhibitors (SGLT2i]); Group B included 77 patients who were treated with at least one of the four pillars before admission.

Demographic data such as age and gender were collected for all participants. Upon admission, patients were classified according to the New York Heart Association’s (NYHA) functional classification system. Left ventricular ejection fraction (LVEF) was assessed using the echocardiographic Simpson method. At admission, patients’ levels of N-terminal pro B-type natriuretic peptide (NT-proBNP), serum sodium, potassium, and creatinine were recorded. During hospitalization, patients in Group A who had not previously received guideline-directed medical therapy began treatment with the four pillars of heart failure therapy. Clinical outcomes were measured, including weight change, blood pressure measurements, and heart rate monitoring. The study also evaluated short-term mortality rates within 30 days post-admission.

The study was conducted in accordance with the principles outlined in the Declaration of Helsinki, ensuring ethical standards for research involving human subjects. Approval was obtained from the Institutional Review Board (IRB) of the Arad Emergency County Hospital (Protocol 41/21 August 2021). Informed consent was obtained from all the patients included in the study.

### 2.2. Statistical Analysis

Continuous variables with non-normal distribution were expressed as the median with their corresponding 95% confidence interval and compared using the non-parametric Mann–Whitney test. Categorical variables were compared using the Chi-squared test. Multivariate regression analyses were employed to adjust for potential confounders and assess the independent effects of treatment on outcomes. A *p*-value of less than 0.05 was considered statistically significant.

## 3. Results

A total of 203 patients with heart failure with reduced ejection fraction (HFrEF) were included in the study and divided into two groups based on their medication regimens before admission. Group A consisted of 126 patients presenting with decompensated heart failure who were not receiving any of the four pillars of heart failure treatment. Group B included 77 patients treated with at least one of the four pillars prior to admission. The demographics and clinical parameters are presented in Table 1.

The median age of patients in Group A was 64 years (95% CI [61.46; 67.54]) compared to 64 years (95% CI [60.8; 67.2]) in Group B, with no statistically significant difference between the two groups (*p* = 0.7405). Regarding gender distribution, 68.25% of patients in Group A were male (86/126), while Group B had a higher proportion of males at 81.81% (63/77), although this difference was not statistically significant (*p* = 0.185).

Upon admission, the distribution of patients in Group A by NYHA class was as follows: 1.19% were classified as NYHA class II, 35.71% as NYHA class III, and 61.90% as NYHA class IV. At discharge, the proportions regarding NYHA classification in Group A changed: 2.38% class I, 61.9% class II, and 30.95% class III.

At the time of admission, the distribution of patients in Group B by NYHA class was the following: 47.22% were classified as NYHA class III, while 52.77% were classified as NYHA class IV. At discharge, the proportions regarding NYHA classification in Group B also changed: 55.55% class II and 44.44% class III. There was a significant decrease in New York Heart Association (NYHA) classification following optimal medical treatment, indicating an improvement in functional capacity as indicated by a decrease in New York Heart Association (NYHA) classification following optimal medical treatment (*p* < 0.001, χ^2^ squared test).

Patients in Group A exhibited higher systolic and diastolic blood pressure levels at admission compared to discharge, with an average reduction of 20 mmHg in systolic blood pressure (admission: 140 mmHg, 95%CI [135.94; 144.06]; discharge: 120 mmHg, 95%CI [116.92; 123.08], *p* < 0.001), and an average reduction of 10 mmHg in diastolic blood pressure (admission: 80 mmHg, 95%CI [77.05; 82.95]; discharge: 70 mmHg, 95%CI [67.71; 72.29], *p* < 0.001). Among patients who had already initiated at least one of the four pillars of heart failure treatment prior to admission (Group B), there was a statistically significant decrease in both systolic and diastolic blood pressure (Group A—admission SBP: 137.5 mmHg, 95%CI [129.8; 145.2]; discharge SBP: 120 mmHg, 95%CI [112.85; 127.15], *p* = 0.02; Group B—admission DBP: 82.5 mmHg, 95%CI [77.6; 87.4]; discharge DBP: 75 mmHg, 95%CI [69.23; 80.77], *p* = 0.0127).

Group A demonstrated a more significant reduction in SBP, with an average decrease of 20 mmHg (95% CI [16.70; 23.29]) compared to a reduction of 15.5 mmHg in Group B (95% CI [9.06; 21.93]). However, this difference was not statistically significant (*p* = 0.24).

The decrease in DBP was similar between the two groups. Group A experienced a reduction of 10 mmHg (95% CI [7.65; 12.34]), while in Group B, the DBP also decreased by 10 mmHg (95% CI [4.65; 15.35]) with no significant difference observed (*p* = 0.74).

In Group A, the median heart rate (HR) decreased from 97 beats per minute (95%CI [91.44; 102.56]) at admission to 76 beats per minute (95%CI [73.36; 76.84]) at discharge (*p* < 0.001). The median heart rate in Group B also showed a significant reduction during hospitalization (admission HR: 97 bpm, 95%CI [89.12; 104.88]; discharge HR: 74 b/min, 95%CI [70.31; 77.69], *p* < 0.001). Group B showed a more substantial decrease in heart rate, with an average reduction of 27 beats per minute (b/min) (95% CI [20.18; 33.81]) compared to a reduction of 20 b/min in Group A (95% CI [15.44; 24.55]). This difference was not statistically significant (*p* = 0.20).

Regarding body weight change, Group A lost an average of 4.5 kg (95% CI [2.22; 4.77], weight at admission: 84.5 kg, 95%CI [80.1–88.9] vs. weight at discharge: 80 kg, 95 %CI [75.82–84.18], *p* = 0.2241), and Group B lost an average of 3 kg, 95% CI [1.21; 4.78]; weight at admission: 87 kg, 95% CI [84.03; 90.97] vs. weight at discharge: 84 kg, 95% CI [77.84; 90.16], *p* = 0.273).

When renal function was evaluated, the serum creatinine levels did not show a statistically significant increase during the initiation of the four pillars treatment; creatinine levels were 1.11 mg/dL (95%CI [1.02; 1.18] at admission and 1.14 mg/dL (95%CI [1.06; 1.22]) at discharge for Group A (*p* = 0.4728), and for Group B, creatinine levels were 1.08 mg/dL, 95%CI [0.95; 1.21] at admission, while creatinine at discharge was 1.13 mg/dL, 95%CI [0.99; 1.27], *p* = 0.4527). Significant differences were observed in creatinine dynamics (Δcreatinine), with Group B showing a higher increase compared to Group A. Group B had an increase of 0.2 mg/dL (95% CI [0.15; 0.24]), while Group A had an increase of 0.1 mg/dL (95% CI [0.07; 0.12]), resulting in a statistically significant difference (*p* = 0.02).

**Figure 1:**
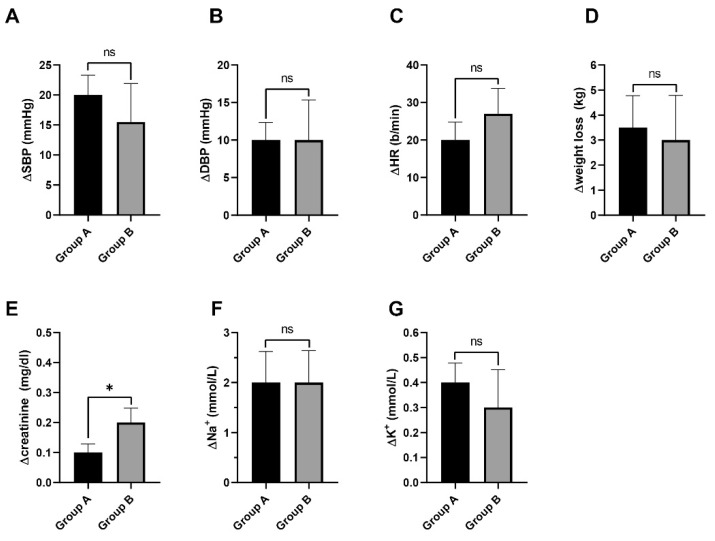
(**A**) A greater decrease in systolic blood pressure was noted in Group A compared to Group B. However, there were no statistically significant differences (*p* = 0.24). (**B**) Groups A and B expressed a similar decrease in DBP with no statistically significant differences (*p* = 0.74). (**C**) Group B experienced a greater decrease in heart rate, but the difference was not statistically significant (*p* = 0.20). (**D**) Both groups experienced weight loss after initiating/adjusting the HF treatment based on the four pillars, with a greater decrease in weight in Group A. However, the differences were not statistically significant (*p* = 0.54). (**E**) Group B experienced a higher increase in serum creatinine levels compared to Group A with statistically significant differences (*p* = 0.02). (**F**) The serum sodium levels remained relatively constant in both groups while initiating/adjusting the HF treatment with no statistically significant differences (*p* = 0.94). (**G**) Both groups experienced a minor increase in the serum potassium levels, but without statistically significant differences (*p* = 0.19). ns—not significant; *—*p* < 0.05.

**Figure 2:**
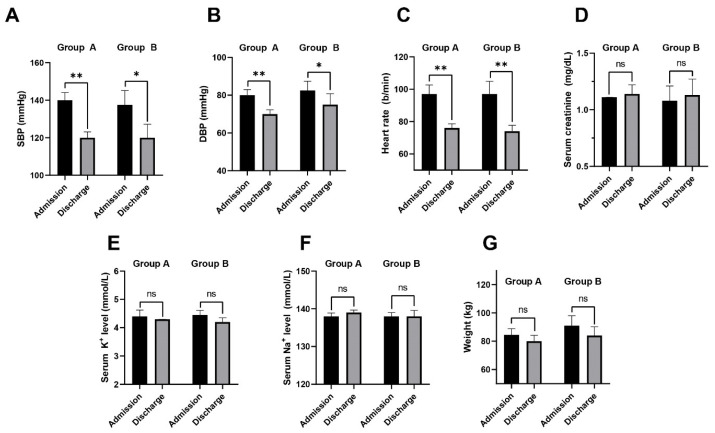
(**A**) Comparison of SBP (mmHg) at admission vs. discharge in Groups A and B; a statistically significant difference between admission and discharge was observed in both cohorts; (**B**) A statistically significant decrease in DBP (mmHg) was observed between admission and discharge in both groups; (**C**) The heart rate (b/min) decreased significantly between admission and discharge in both groups due to initiation/adjustment of the four pillar treatment; (**D**) The serum creatinine levels (mg/dL) remained relatively constant throughout hospitalization in both groups; (**E**) Serum Na^+^ (mmol/L) did not manifest any significant changes throughout hospitalization in both groups; (**F**) Serum K^+^ (mmol/L) remained relatively constant in both groups; (**G**) Group A experienced a higher decrease in body weight than Group A, but without statistical significance; ns—not significant; *—*p* < 0.05; **—*p* < 0.01.

No significant differences were noted between the groups regarding plasma sodium dynamics, with both groups showing an average decrease of 2 mmol/L (Group A: 95% CI [1.36; 2.63], Group B: 95% CI [1.35; 2.64], *p* = 0.96). Similarly, potassium changes were similar, with Group A increasing by 0.4 mmol/L (95% CI [0.32; 0.47]) and Group B by 0.5 mmol/L (95% CI [0.14; 0.45]), with no significant differences (*p* = 0.19). Electrolyte levels did not express notable changes: serum sodium concentration. Simultaneously, potassium levels remained relatively stable, with values of 4.4 mmol/L at admission (95% CI [4.28; 4.52]) compared to 4.3 mmol/L (95%CI [4.21; 4.39]) at discharge (*p* = 0.2806). The comparative analysis is illustrated in Figure 1 and Figure 2. 

The left ventricular ejection fraction (LVEF) measurements were comparable between the two groups, with Group A showing an average of 30%, 95%CI [28.76; 31.24] and Group B showing 30%, 95%CI [27.99; 32.01]. The difference was not statistically significant (*p* = 0.76). Similarly, N-terminal pro B-type natriuretic peptide (NT-proBNP) levels at admission were nearly identical between the groups, with Group A having 4487 ng/mL, 95% CI [3983.33; 4990.66] and Group B having 4711.5 ng/mL, 95%CI [3966.86; 5456.13], with no statistical significance (*p* = 0.6305).

**Figure 3:**
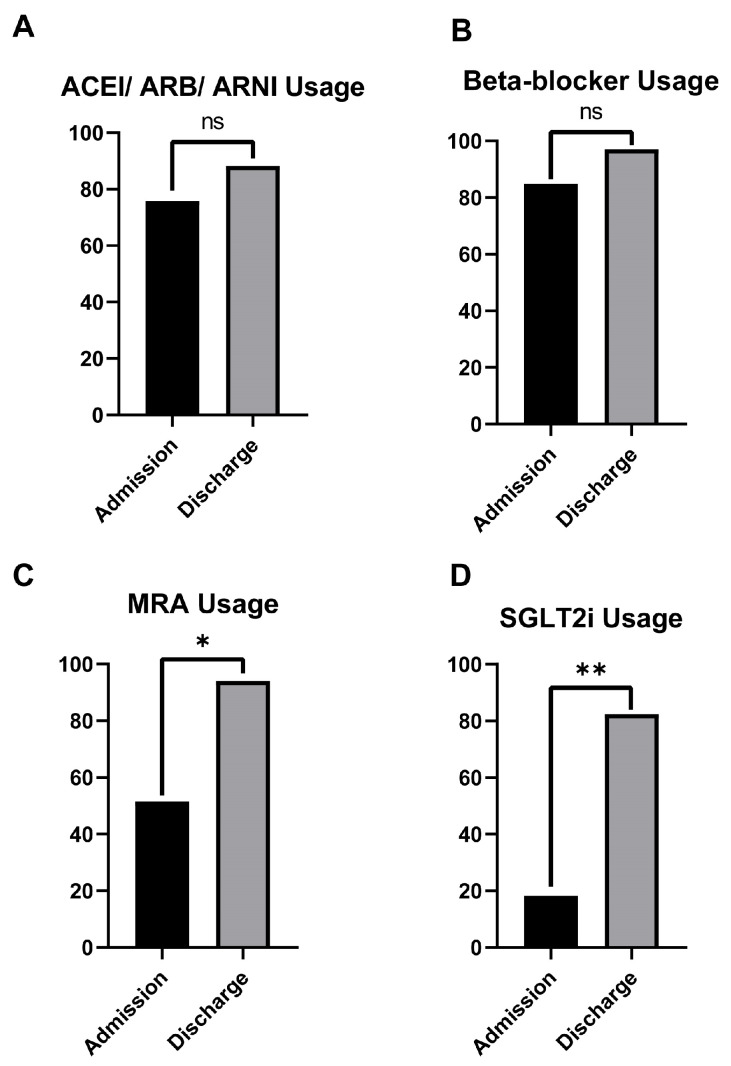
The use of the four pillars of heart failure treatment at admission and at discharge in Group B. Statistically significant differences can be observed with respect to MRA (*p* = 0.01) and SGLT2i usage (*p* < 0.001); *—*p* < 0.05; **—*p* < 0.01; ns—not significant.

Among the 77 patients in Group B with prior heart failure treatment, the baseline medication usage prior to admission was as follows: 75.32% (58/77) were receiving ACE inhibitors, angiotensin receptor blockers, or angiotensin receptor–neprilysin inhibitors (ACEI/ARBs/ARNI); 84.41% (65/77) were on beta-blocker therapy; 51.94% (40/77) were treated with mineralocorticoid receptor antagonists (MRA); and only 18.18% (14/77) had been prescribed SGLT2 inhibitors. During hospitalization, we observed a significant increase in the use of MRAs and SGLT2 inhibitors. Specifically, the percentage of patients receiving ACEI/ARBs/ARNI increased from 75.32% to 88.31% (68/77, *p* = 0.18), beta-blocker therapy increased from 84.41% to 97.40% (75/77, *p* = 0.08), MRA usage increased significantly from 51.94% to 94.80% (74/77, *p* = 0.01), and SGLT2 inhibitor increased significantly from 18.18% to 83.11% (64/77, *p* < 0.001) (Figure 3).

The 30-day mortality rates showed no significant difference between the groups: Group A had a mortality rate of 7.14%, while Group B had a rate of 5.55%, with a *p*-value of 0.74 (χ^2^ test for proportions).

## 4. Discussion

The initiation and optimization of the four pillars of HFrEF treatment (BB, SGLT2i, MRA, ARNI) are essential after an episode of decompensation for the improvement of survival. It is known that, although diuretics improve symptoms in case of decompensation, they do not reduce mortality [9,10,11,12]. The initiation or optimization of treatment is routinely recommended before discharge according to the consensus document released by HFA. Although some trials have shown that high doses are more effective than low doses in the treatment of HFrEF [13,14], the ESC guideline of 2021, updated in 2023 [3], states that it is more important to introduce or optimize the four pillars and titrate from low to optimal doses. Our study also validated this statement. This finding has also been suggested by several authors [12,15,16]. Both at initiation and optimization, we tried to implement the four pillars in the therapeutic regimen as quickly as possible, even at low doses. Then we focused on dose optimization for drugs that required it. In daily clinical practice, the four pillars are often underutilized even when there are no contraindications [17,18,19]. One of the main reasons leading to the underutilization of medication in HFrEF is the fear of adverse effects such as hypotension, hyperkalemia, or deterioration of renal function. Our data was collected in a county hospital. The patients in Group A, despite having a previous diagnosis of HF, were not treated with GDMT. Several other studies published recently suggested that, in the real world, GDMT for patients with HFREF is significantly underused. From this point of view, our study showed that both initiation and optimization of treatment are relatively safe and well-tolerated. Even if the patient initially required closer monitoring of blood pressure, K^+^, Na^+^, and renal function, the initiation/optimization of treatment should not be delayed. Other factors that lead to the underutilization of drugs in HFREF are clinical inertia and treatment nonadherence [20,21]. From our study, we observed that a physician’s inertia in introducing the four pillars is of greater importance than adherence (initially, more patients were off medication or only utilizing one or two pillars). After treatment initiation/optimization, most patients adhered to treatment. Although under clinical trial conditions, adherence is higher than in real life, we consider physician inertia to be more important and worth every effort to overcome.

In our study, both groups of patients exhibited similar demographic characteristics, particularly in terms of age and gender distribution. This similarity is important, as it suggests that the cohorts are comparable, allowing for a more accurate evaluation of treatment effects and outcomes. Furthermore, both groups presented with comparable levels of cardiac function and heart failure severity, which reinforces the validity of comparing their clinical outcomes in-hospital and post-hospitalization. Similarly, the study published by Sljivo et al. revealed that the majority of patients who presented for acute heart failure decompensation were males with histories of hypertension, dyslipidemia, and smoking, as well as high levels of NT-proBNP at admission [22]. The analysis revealed that short-term survival outcomes were similar for both groups following hospitalization. This finding is significant, as it suggests that the interventions applied during hospitalization were effective regardless of the initial severity of heart failure and their prior treatment.

A notable observation was the decrease in heart rate during hospitalization, likely attributed to the initiation of beta-blocker therapy and adjustments in fluid status. This decrease indicates effective symptom management and stabilization of cardiac function. This is important, as it is well known that a high resting heart rate is a poor prognostic factor in heart failure [23,24].

Initially low systolic BP has often been considered a factor impeding the initiation or optimization of the four pillars, especially ARNI. In our study, no statistically significant differences in BP between the two groups were found, and this did not impede initiating or optimizing treatment. In the CHAMP-HF registry [25], it was reported that less than 20% of patients had all four pillars implemented, which was not correlated with initial BP, with implementation remaining low even if SBP >110 mmHg. The findings underscore the importance of initiating comprehensive heart failure therapy during hospitalization, which has significantly improved patient outcomes.

The analysis focused on patients receiving at least one of the four pillars of heart failure therapy during hospitalization revealed several significant changes in clinical parameters. These changes included improvements in weight, BP control, and increased functional capacity without adversely affecting renal function or electrolyte balance. The stability of renal function suggests that aggressive heart failure management can be safely implemented without adversely affecting homeostasis.

The results further highlight the role of enhanced pharmacotherapy for heart failure during hospitalization, particularly regarding the use of mineralocorticoid receptor antagonists (MRAs) and SGLT2 inhibitors. These medications have been associated with improved outcomes, emphasizing their importance in heart failure management protocols along with BB and ARNI.

Lastly, the high number of patients diagnosed with heart failure (HF) who are admitted without effective treatment is concerning. Efforts should be made to further increase awareness of the importance of HF treatment in ambulatory settings to prevent hospitalizations and frequent decompensations and to improve survival rates.

## 5. Conclusions

The initiation and optimization of comprehensive GDMT during hospitalization in patients with HFREF improves their outcomes. The stability of paraclinical parameters, coupled with notable improvements in clinical metrics such as weight loss, blood pressure control, and heart rate reduction, highlights the effectiveness of current treatment strategies. The initiation of the four pillars of treatment in HFREF during hospitalization is achievable, safe, and well-tolerated.

## Figures and Tables

**Table 1:** Demographics, clinical and biological parameters of patients in Group A (without any of the four pillars of heart failure treatment) compared to Group B (with at least one of the four pillars). A statistically significant difference was found in terms of alcohol consumption (greater in Group A) and a greater proportion of males in Group A.

	Group A (*n* = 126)	Group B (*n* = 77)	
Age (years)	64, 95% CI [61.46–67.54]	64 (95% CI [60.8–67.2])	*p* = 0.7405
Gender M (n, %)	86 (68.25%)	63 (81.81%)	*p* = 0.0343
F (*n*, %)	40 (31.75%)	15 (18.19%)	*p* = 0.053
Weight (kg)	84.5, 95% CI [80.1–88.9]	91 (95% CI [84.03; 97.97])	*p* = 0.464
Alcohol consumption (*n*, %)	17, 16.5%	4, 5.1%	*p* = 0.01
Smoking history (*n*, %)	27, 21.4%	12, 15.55%	*p* = 0.30
Hypertension (*n*, %)	49, 39.1%	27, 35.1%	*p* = 0.56
Ischemic heart disease (*n*, %)	33, 26.2%	25, 32.4%	*p* = 0.3403
Valvular heart disease (*n*, %)	20, 15.9%	17, 22.1%	*p* = 0.2683
SBP (mmHg)	140, 95% CI [135.94; 144.06]	137.5, 95% CI [129.8; 145.2]	*p* = 0.787
DBP (mmHg)	80, 95% CI [77.05; 82.95]	82.5, 95% CI [77.6; 87.4]	*p* = 0.89
HR (b/min)	97, 95% CI [91.44–102.56]	97, 95% CI [89.12–104.88]	*p* = 0.835
Atrial fibrillation (*n*, %)	46, 36.5%	38, 49.4%	*p* = 0.07
QRS duration (ms)	114.5, 95% CI [107.5–121.44]	117, 95% CI [107.81–126.19]	*p* = 0.367
LVEF (%)	30%, 95% CI [28.76; 31.24]	30%, 95% CI [27.99; 32.01]	*p* = 0.76
NT-proBNP (ng/mL)	4487, 95% CI [3983.33; 4990.66]	4711.5, 95% CI [3966.86; 5456.13]	*p* = 0.63
Creatinine (mg/dL)	1.11, 95% CI [1.04–1.18]	1.08, 95% CI [0.95–1.21]	*p* = 0.78
Glycemia (mg/dL)	121, 95% CI [105.77–136.23]	152, 95% CI [120.9–184.1]	*p* = 0.245
AST (IU/L)	31.5, 95% CI [22.71–40.29]	27, 95% CI [16.1–37.9]	*p* = 0.288
ALT (IU/L)	37, 95% CI [20.67–53.33]	21, 95% CI [13.99–28.01]	*p* = 0.08
Serum Na^+^ (mmol/L)	138 (95% CI [136.18; 138.92])	138, 95% CI [136.99; 139.01]	*p* = 0.772
Serum K^+^	4.4, 95% CI [4.28; 4.52]	4.45, 95% CI [4.26; 4.61]	*p* = 0.662

## Data Availability

The original contributions presented in this study are included in the article. Further inquiries can be directed to the corresponding author.
